# Supplementary material for: Iridescent Features Correlating with Periodic Assemblies in Custom-Crystallized Arylate Polyesters
Source: Int J Mol Sci. 2023 Oct 24;24(21):15538. doi: 10.3390/ijms242115538 (PMC10650520; doi:10.3390/ijms242115538)
Supplement: Supplementary file 1 [file ijms-24-15538-s001.zip › ijms-2669554-supplementary.pdf]

Supporting Information

Iridescent Features Correlating with Periodic Assemblies in Custom-Crystallized Arylate Polyesters

Widyantari Rahmayanti, Selvaraj Nagarajan, Ya-Sen Sun * and Eamor M. Woo *

Department of Chemical Engineering, National Cheng Kung University, No. 1, University Road, Tainan 701-01, Taiwan; widyantarii32@gmail.com (W.R.); nagarajan.tech@gmail.com (S.N.)

* Correspondence: yssun@gs.ncku.edu.tw (Y.-S.S.); emwoo@mail.ncku.edu.tw (E.M.W.); Tel.: +886-6-275-7575 (ext. 62670) (E.M.W.); Fax: +886-6-234-4496 (E.M.W.)

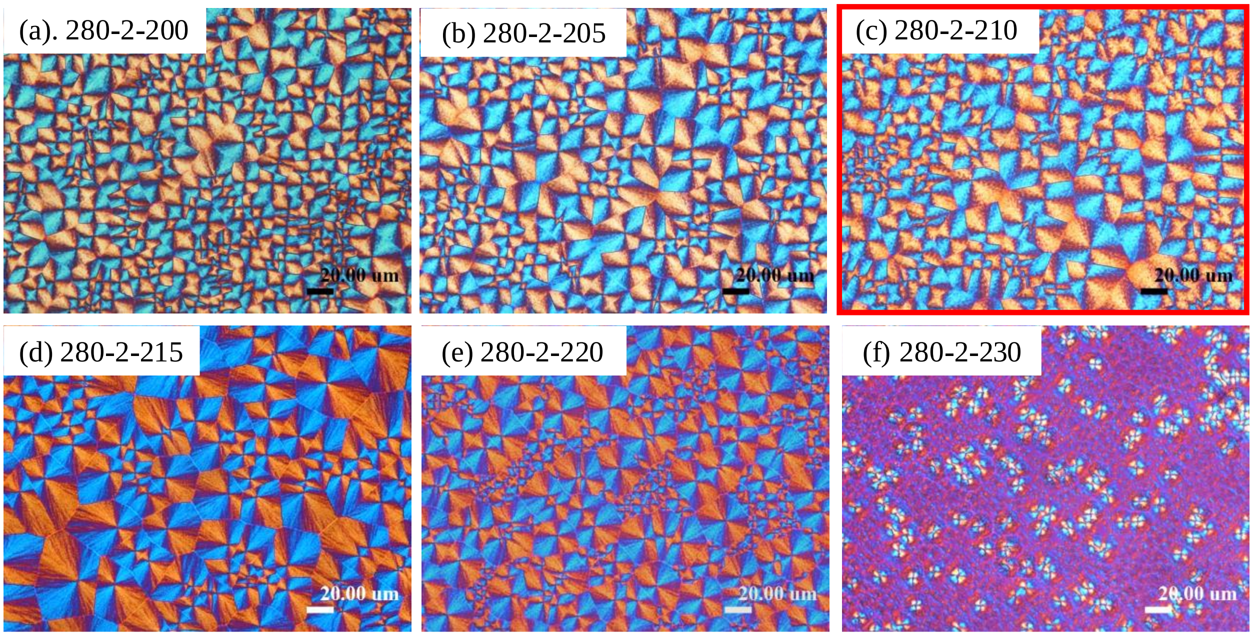

Figure S1. POM images for PET films crystallized at Tc=200, 205, 210, 215, 220, 230 °C, after being melted for 2 minutes at a same max-melt temperature (T_{max}) of 280 °C.
